# Implementation, Dosimetric Assessment, and Treatment Validation of Knowledge-Based Planning (KBP) Models in VMAT Head and Neck Radiation Oncology

**DOI:** 10.3390/biomedicines11030762

**Published:** 2023-03-02

**Authors:** Anna-Maria Fanou, Georgios Patatoukas, Marina Chalkia, Nikolaos Kollaros, Andromachi Kougioumtzopoulou, Vassilis Kouloulias, Kalliopi Platoni

**Affiliations:** 1 Medical Physics Unit, Second Department of Radiology, Medical School, National and Kapodistrian University of Athens, Attikon University Hospital, Haidari, 12462 Athens, Greece; 2 Radiation Therapy Unit, Second Department of Radiology, Medical School, National and Kapodistrian University of Athens, Attikon University Hospital, Haidari, 12462 Athens, Greece

**Keywords:** knowledge-based planning, RapidPlan™, head and neck cancer, plan deliverability

## Abstract

The aim of this study was to evaluate knowledge-based treatment planning (KBP) models in terms of their dosimetry and deliverability and to investigate their clinical benefits. Three H&N KBP models were built utilizing RapidPlan™, based on the dose prescription, which is given according to the planning target volume (PTV). The training set for each model consisted of 43 clinically acceptable volumetric modulated arc therapy (VMAT) plans. Model quality was assessed and compared to the delivered treatment plans using the homogeneity index (HI), conformity index (CI), structure dose difference (PTV, organ at risk—OAR), monitor units, MU factor, and complexity index. Model deliverability was assessed through a patient-specific quality assurance (PSQA) gamma index-based analysis. The dosimetric assessment showed better OAR sparing for the RapidPlan™ plans and for the low- and high-risk PTV, and the HI, and CI were comparable between the clinical and RapidPlan™ plans, while for the intermediate-risk PTV, CI was better for clinical plans. The 2D gamma passing rates for RapidPlan™ plans were similar or better than the clinical ones using the 3%/3 mm gamma-index criterion. Monitor units, the MU factors, and complexity indices were found to be comparable between RapidPlan™ and the clinical plans. Knowledge-based treatment plans can be safely adapted into clinical routines, providing improved plan quality in a time efficient way while minimizing user variability.

## 1. Introduction

Intensity-modulated arc therapy (IMRT) and volumetric Modulated Arc Therapy (VMAT) have become standard treatment approaches especially for complex irradiation geometries such as the ones found in head and neck (H&N) carcinoma, in which a large number of organs at risk (OARs) are close to the target volume [1]. In inverse treatment planning, an optimization problem must be solved. The optimization problem involves several constraints which must be fulfilled and an objective function that quantifies the treatment plan quality [2]. The treatment planner is the one who steers the optimization engine by searching for the trade-offs between applying an adequate dose to the tumor and sparing the surrounding normal tissue [3]. Therefore, inverse treatment planning can be a challenging trial and error process, and it is subjected to inter-planner variability as it involves critical thinking [2].

Automation has gained interest in radiotherapy because it exploits state-of-the-art technology and computer power to minimize inter-planner variability and improve plan quality [4]. Knowledge-based planning (KBP) is considered an example of an automated treatment planning solution in radiotherapy [5]. Knowledge is captured from libraries of high-quality treatment plans to train a model which predicts the range of the dose volume histograms (DVHs) for the OARs (DVH estimates) for any new patient with similar anatomical features. KBP is considered a part of automated treatment planning solutions, as it automatically places optimization objectives, called line objectives, to predict the DVH for the OARs. A prevalent commercial KBP tool is RapidPlan™ (Varian Medical Systems), which was employed in this work.

There have been several studies considering various RapidPlan™-related aspects starting from its efficacy in reducing inter-planner variability to its use as a quality assurance (QA) tool, especially for H&N carcinoma [6,7,8]. However, to our knowledge, no studies focus on plan deliverability, a complexity assessment of RapidPlan™ plans, or their correlation with H&N cancer cases. There is one work studying plan deliverability as a part of an investigation of dose escalation according to the gross tumor volume (GTV) in poor-prognosis oropharyngeal squamous cell cancer cases, combined with RapidPlan™ and multi-criteria optimization (MCO) [9]. However, Grocutt et al. [9] conducted a dose escalation study re-optimizing all clinical plans with RapidPlan™ and MCO, so a comparison of plans optimized with and without RapidPlan™ was out of the scope of their study.

The aim of this study was to compare treatment plans optimized with RapidPlan™ with the clinical ones and to investigate the potential benefit of the introduction of RapidPlan™ in clinical routines. To the best of our knowledge, this is the first study that, in addition to focusing dosimetry, focuses on a plan deliverability evaluation of the RapidPlan™ KBP tool for H&N cases, explores the factors which may affect plan complexity, and investigates a possible correlation between them.

## 2. Materials and Methods

### 2.1. Patient Selection

The high-quality clinically acceptable treatment plans for 65 patients with H&N cancer who were treated with the VMAT technique between March 2019 and October 2021 in our department were included in this study. The H&N cancer patients were classified as larynx, nasopharynx, and oropharynx cases according to the guidelines of the radiation oncologists in our department. Three RapidPlan™ models were created based on the dose prescriptions for the planning target volumes (PTV), namely 54 Gy, 60 Gy, and 70 Gy (using 2 Gy per fraction). A total of 43 patients (129 treatment plans in total) was considered the input for the training process of the RapidPlan™ models (model creation). The closed-loop validation set consisted of 10 patients out of the ones in the training set. The remaining 22 patients (66 treatment plans in total) were considered in the open-loop validation set. A commercial KBP tool named RapidPlan™, released in 2014 by Varian Medical Systems, and compatible with version 15.1 of the eclipse treatment planning system (TPS) available at our institution, was used to generate the H&N models, according to manufacturer guidelines [10]. RapidPlan™ is a KBP tool that uses machine learning to consider a patient’s anatomy and planning goals. As an output, it provides the user with a prediction of the DVH for the OARs and the optimization objectives for any new patient with similar anatomical features to those patients used for model training.

### 2.2. Model Creation

To create each model, the selected VMAT treatment plans from our institution’s database were uploaded into the model configuration module of the RapidPlan™ software. Each OAR structure from each uploaded plan was matched with the corresponding OAR code from the RapidPlan™ database. The RapidPlan™ algorithm splits each OAR into the following segments: the out-of-filed region (where no primary radiation is received by this part of the OAR, only a scattered dose), the leaf transmission region (where the radiation passes through the closed multi-leaf collimator (MLC) leaves, and where the jaws do not stop this radiation), the in-field region (where a part of the OAR is located inside the primary beam), and the target overlap region (where a part of the OAR overlaps with the PTV). RapidPlan’s™ algorithm (a DVH estimation algorithm) uses principal component analysis and regression methods to predict the DVH and the optimization objectives for the OARs in order to train the model [10].

**Figure 1:**
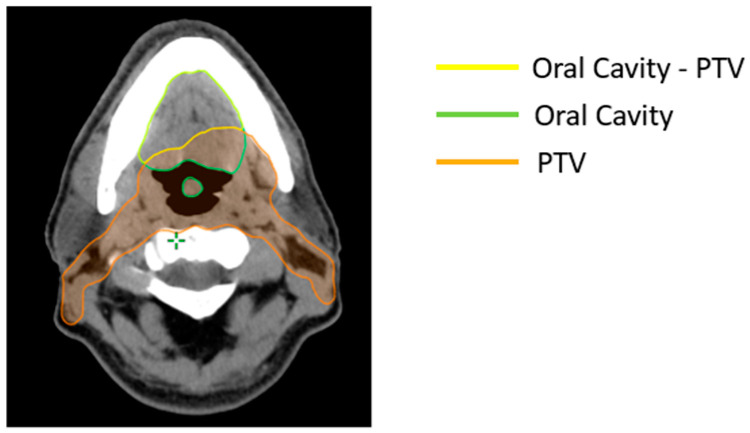
Boolean structure: oral cavity minus region overlapping with the planning target volume (PTV).

According to manufacturer suggestions, the matched OARs should not overlap with the PTV. As a result, 453 Boolean structures were created to exclude the overlap region, for all patients included in the training set (Figure 1). Therefore, the non-involved region of OARs was considered during optimization with the RapidPlan™ models.

### 2.3. Model Verification

Once the creation of the RapidPlan™ model was complete, a model verification was conducted to assess the quality of the RapidPlan™ model from a mathematical/statistical point of view. A model’s goodness-of-fit may be compromised by plans which are considered outliers. The potential outliers were identified using statistical parameters, originating from the training phase of the RapidPlan™ models.

The potential outliers were classified as follows: influential points, geometric outliers, and dosimetric outliers. The influential point (treatment plan) pulls along the regression line, and the model does not represent the majority of the training data. The geometric outliers are treatment plans in which one or more OAR structures have different geometrical features compared to the rest of the training plans. The dosimetric outliers are treatment plans in which one or more OAR structures differ dosimetrically from the other treatment plans. The dosimetric outliers may be divided into two categories positive dosimetric outliers and negative dosimetric outliers [11]. Positive dosimetric outliers are the treatment plans whose clinical DVH is better than the predicted DVH estimate from RapidPlan™. The negative dosimetric outliers are the treatment plans whose clinical DVH is worse than the predicted DVH estimate from RapidPlan™. The implementation guide of RapidPlan™ recommends the re-planning of the negative dosimetric outliers [10]. However, re-planning negative dosimetric outliers is a very time-intensive process, and it was not implemented in this study.

**Figure 2:**
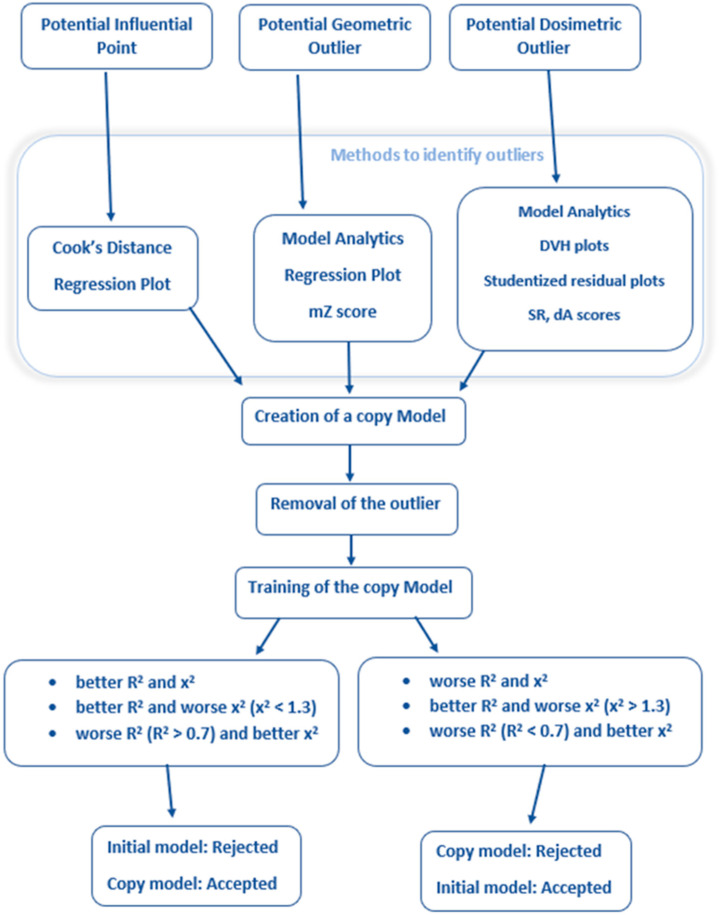
Workflow followed for investigation of outliers.

As a double check, the Model Analytics software was utilized. Model Analytics (Varian Medical Systems) is a cloud application in which the user can upload a RapidPlan™ model to get statistical information and recommendations about the potential outliers. Figure 2 summarizes the workflow applied in this study for the investigation of the outliers.

### 2.4. Model Validation

Model Validation indicated if the model could be applied in clinical practice. The validation consisted of two steps: closed-loop and open-loop validation. Closed-loop validation aims to test the reproducibility of the RapidPlan™ model. During closed-loop validation, some of the plans used for model’s training were re-optimized with RapidPlan™ models and compared to the clinical ones. During open-loop validation, the plans not included in the training set were re-optimized with RapidPlan™ models and compared to the clinical ones. Open-loop validation aims to check a model’s ability to predict accurately the DVH and the optimization objectives for any new patient.

A copy of the clinical plan was created for each treatment plan to keep the beam configuration constant. The copy of the clinical plan was re-optimized with the correspondingly trained RapidPlan™ model, depending on dose prescription.

Firstly, clinically acceptable RapidPlan™ optimized plans with a single RapidPlan™ -based optimization, without any intervention from the planner in the optimization objectives, were created. If the first optimization was insufficient to create a clinically acceptable plan, a second optimization with the RapidPlan™ model was performed, where the planner could modify the optimization objectives.

At the beginning of the validation process, a template with the optimization objectives and the corresponding priorities should be defined for each KBP model. The optimization objective templates were created based on the quantitative analysis of normal tissue effects in the clinic (QUANTEC) dose constraints. At first, three templates were created for the optimization objectives, one for larynx cases, one for nasopharynx cases, and one for oropharynx cases, but to conclude to the three final templates, a refinement of the optimization objectives and iterative checks on a group of patients took place, in accordance with the literature [12,13].

Both the clinical- and RapidPlan™-optimized plans of the validation set were generated with Eclipse TPS version 15.1 (Varian Medical Systems) and the photon optimizer (PO) available at our department. Version 15.1.51 of the anisotropic analytical algorithm (AAA) was adopted for the dose calculation with a dose resolution grid of 2.5 mm. One to four VMAT arcs with a 6 MV beam were used to create the clinical and RapidPlan™ treatment plans. VitalBeam linear accelerators (Varian Medical Systems) were used to deliver the plans for the validation process. All the clinical plans were approved by a radiation oncologist and a medical physics expert, according to our institution’s protocols.

### 2.5. Dosimetric and Plan Deliverability Evaluation

Dose statistics derived from the DVHs were analyzed to study if the RapidPlan™-optimized plans satisfy the OAR dose constraints and the desired coverage of the PTV. The dose statistics for the OARs were extracted from the DVHs of the plan sum. Concerning the dosimetric evaluation of the PTV, the homogeneity index (HI), conformity index (CI), and the volume of the PTV covered by 107% of the prescription isodose curve ($V_{107\%}$) were recorded.

HI is defined as shown in Equation (1):

(1) $$\mathrm{HI}=\frac{D_{2\%}-D_{98\%}}{D_{p}}$$

where $D_{2\%}$ is the near-maximum dose, $D_{98\%}$ is the near-minimum dose, and $D_{P}$ is the prescription dose received by the PTV.

CI is defined as shown in Equation (2):

(2) $$\mathrm{CI}=\frac{V_{95\%}}{V_{\mathrm{PTV}}}$$

where $V_{95\%}$ is the volume of the PTV covered by 95% of the prescription isodose level, and $V_{\mathrm{PTV}}$ is the volume of the PTV.

The number of monitor units (MU) per plan, the modulation factor—MU factor (Equation (3))—and the complexity index [14,15] (Equation (4)) were evaluated. The MU was extracted from the TPS of the corresponding treatment plan, and the complexity index was calculated using a script. The code of the complexity script utilized in this study was written in Python and it was used in the work of Younge et al. [15]. The DICOM RT plan files exported from the TPS for each plan were uploaded to the complexity script to derive the complexity indices. Moreover, Python and Anaconda 3 were used to run the script.

The MU factor is defined as shown in Equation (3):

(3) $$\mathrm{MU}\;\mathrm{factor}=\frac{\mathrm{Monitor}\;\mathrm{Units}\;\left(\mathrm{MUs}\right)\;\mathrm{per}\;\mathrm{phase}}{\mathrm{Dose}\;\mathrm{prescription}\;\mathrm{per}\;\mathrm{phase}}$$

The complexity index is defined as shown in Equation (4):

(4) $$\mathrm{complexity}\;\mathrm{index}=\frac{1}{\mathrm{MU}}\sum_{i=1}^{N}{\mathrm{MU}}_{i}\times \frac{y_{i}}{A_{i}}\left[{\mathrm{mm}}^{-1}\right]$$

where MU is the total number of monitor units in the plan, ${\mathrm{MU}}_{i}$ is the number of monitor units delivered through aperture i, the sum of Σ comprises all the control points from 1 to N, $A_{i}$ is the open area of aperture I, and $y_{i}$ is the aperture perimeter excluding the MLC leaf ends [14].

The complexity index is a robust metric against the dose in the plan and is relatively independent of the treatment volume [14].

Regarding the dosimetric assessment of the OARs, 18 OARs were evaluated in this study. The relative difference (Δrelative) between the clinical and the RapidPlan™ plans for these OARs is defined as shown in Equation (5):

(5) $$\Delta \mathrm{relative}=\left(\frac{\mathrm{clinical}-\mathrm{RapidPlan}}{\mathrm{clinical}}\right)\;x\;100\%$$

A Wilcoxon signed-rank test was used for the statistical analysis.

Patient-specific quality assurance (PSQA) is highly recommended by Task Group 218 (TG-218) of the American Association of Physicists in Medicine (AAPM) [16] to ensure that the dose delivered to the patient is in accordance with the dose distribution calculated by the TPS. In this work, PSQA was performed with ArcCHECK™ phantom [17] (Sun Nuclear Corporation), and the obtained 2D gamma passing rates (2D GPRs (%)), using the gamma criteria 3%/3 mm, 3%/2 mm, and 2%/3 mm for the RapidPlan™-optimized plans were compared with those of the clinical ones.

The Wilcoxon signed-rank test was used to check if the differences in the 2D GPRs (%), MUs, MU factor, and the complexity index between the RapidPlan™ and clinical plans were statistically significant. A Spearman’s rho correlation coefficient was utilized to determine if there was a correlation between the complexity index and MUs or the MU factor and if there was also a correlation between the 2D GPRs (%) and the complexity index, the Mus, or the MU factor.

## 3. Results

### 3.1. RapidPlan’s™ Success and Failure Rates

RapidPlan’s™ success and failure rates, provided in Table 1, defined RapidPlan’s™ ability or lack thereof to create a clinically acceptable treatment plan. In 54.5% of the open-loop set of plans, a clinically acceptable plan was feasible with the first optimization, without the intervention of the treatment planner. Upon the first optimization of the open-loop set, failure was observed for 83.3% of the plans belonging to the nasopharynx treatment site. For the closed loop, in 80% of plans a clinically acceptable treatment plan was feasible with the first optimization. During the second optimization (where the planner adapted the optimization objectives), RapidPlan’s™ success rate was at least 90% for both the closed- and the open-loop sets.

### 3.2. Dosimetric Evaluation

The results from the comparison (relative differences) between the clinical and RapidPlan™ plans for the open-loop validation set are provided in Table 2. For the low-risk PTV, the HI, CI, and $V_{107\%}$ showed no statistically significant difference. For the intermediate-risk PTV, $V_{107\%}$ was 2.68%, which is significantly better for RapidPlan™, while the CI was 3%, which is significantly improved for the clinical plan. For the high-risk PTV, the CI and HI were comparable, but $V_{107\%}$ was 8.73%, which is significantly worse for the RapidPlan™ plans.

Table 3 shows the relative difference between the clinical plans and the RapidPlan™-generated plans for the 18 OARs assessed in this work.

The RapidPlan™-generated plans achieved statistically significant lower doses in 58.3% of the OARs. For example, the maximum quantity for the brainstem, the right lens, as well as $V_{54\mathrm{Gy}}$ for the esophagus improved significantly above 14% with RapidPlan™. As a result, RapidPlan™ performed equally to or better than the clinical plan for the dosimetric endpoints of the OAR structures evaluated in this study.

### 3.3. Plan Deliverability

The results for the mean 2D GPRs (%) of the open-loop validation set are presented in Table 4. For the 3%/3 mm criterion, RapidPlan’s™ mean 2D GPRs (%) were higher than the clinical ones for phase 70 Gy (99% versus 98%), while they showed no statistically significant difference in phases 54 Gy and 60 Gy. For the 3%/2 mm and 2%/3 mm criteria, RapidPlan’s™ mean 2D GPRs (%) were higher for phases 60 Gy and 70 Gy, while they were comparable for phase 54 Gy.

Regarding MUs, the MU factor, and the complexity index, for the open-loop validation group, the plans showed no statistically significant difference (Table 5).

The complexity index for both clinical and RapidPlan™ generated plans was found to be weakly to moderately correlated with the MUs and the MU factor for phases 54 Gy and 60 Gy, and no correlation was observed for phase 70 Gy.

**Figure 3:**
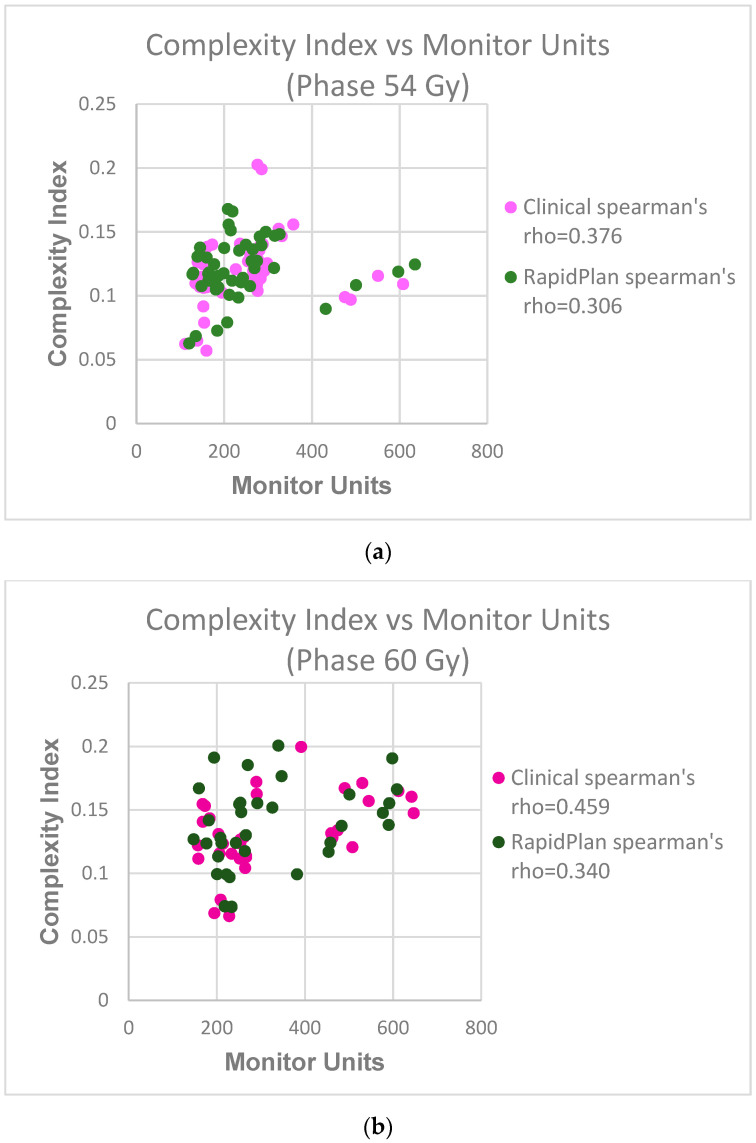
(**a**). Complexity index against monitor units for phase 54 Gy, and for clinical and RapidPlan™ plans in the open-loop validation set. (**b**) Complexity index against monitor units for phase 60 Gy for clinical and RapidPlan™ plans in the open-loop validation set.

In Figure 3a,b, the complexity index is presented against the MUs for phases 54 Gy and 60 Gy of the clinical and RapidPlan™ plans in which a statistically significant correlation was observed. It can be seen that the complexity index is correlated moderately with the MUs for both phases and both plan categories.

For the RapidPlan™ plans, no correlation between the 2D GPRs (%) and the MUs, MU factor, or the complexity index was found. For the clinical plans, a moderate correlation was observed for phase 70 Gy between the 2D GPRs (%) and the MUs, and between the 2D GPRs (%) and the MU factor, applying the 3%/2 mm and 2%/3 mm criteria with Spearman’s rho values of −0.483 (*p*-value = 0.027) and −0.437 (*p*-value = 0.048), respectively.

## 4. Discussion

The introduction of RapidPlan™ H&N models seems to be of clinical importance, since our dosimetry assessment revealed better OAR sparing in 58.3% of the OARs without compromising plan deliverability (with comparable or better 2D GPRs (%)) with comparable MUs and a comparable complexity index. Concerning the PTV dosimetric assessment, for the low-risk PTV, no statically significant difference was observed between the RapidPlan™ and clinical plans. For the intermediate-risk PTV, the CI was better for the clinical plans while V_107%_ was better for the RapidPlan™ plans, and for the high-risk PTV, the CI and HI showed no statically significant difference, but V_107%_ was better for the RapidPlan™ plans. Considering the fact that the H&N are complex treatment sites, the results found in this study are encouraging and show that RapidPlan™ could assist medical physicists in the optimization of treatment plans even in demanding irradiation geometries.

### 4.1. Dosimetric Evaluation

The dosimetric results showed that the sparing of the OARs was better and that the PTV coverage was comparable between clinical and RapidPlan™ plans. Specifically for the OARs, the brainstem, the esophagus, the left parotid gland, and the right parotid gland received lower doses at an average of 3.7 Gy, 7.8 Gy, 1.9 Gy, and 2.3 Gy with RapidPlan™ (a statistically significant outcome). It is worth mentioning that in this study the focus was placed on the open-loop validation results because only open-loop validation will provide the user with the confidence that the model can be applied clinically. Other studies report comparable or improved PTV coverage and OAR sparing with RapidPlan™ for H&N models [13,18,19,20]. Kaderka et al. trained a RapidPlan™ model with 52 H&N patients and found statistically significant lower doses in the left and right cochlea, cricopharyngeus, esophagus, larynx, and the parotid glands [21]. Another study evaluated the dosimetric indices of RapidPlan™ plans on Varian LINACS, and for the H&N model they concluded that the dosimetric indices for the PTV and OARs were comparable regardless of energies and MLC types [22]. Moreover, it has been demonstrated that knowledge-based DVH predictions generated from RapidPlan™ H&N models can be used for plan quality assurance purposes, especially for the plans intended for use in clinical trials [7,8,23,24]. Therefore, the suitability of RapidPlan™ as a QA tool reveals not only the good plan quality achieved via RapidPlan™ but also that accurate DVH predictions (DVH estimates) can be obtained [8].

### 4.2. Plan Deliverability

In this study, for the 3%/2 mm and 2%/3 mm criteria, the 2D GPRs (%) were better for RapidPlan™ compared to the clinical ones for phases 60 Gy and 70 Gy, while a statistically significant difference was not observed for phase 54 Gy. This might be attributed to the fact that the PTV volume is usually bigger and closer to some OARs in phase 54 Gy (low-risk PTV) compared to phases 60 Gy (intermediate-risk PTV) or 70 Gy (high-risk PTV). This fact could make it more difficult during optimization to find the optimal trade-off between PTV coverage and OAR sparing, and it could probably lead to smaller 2D GPRs (%) for phase 54 Gy plans. Moreover, the MUs, the MU factor, and the complexity index for RapidPlan™ were similar to those of the clinical ones. The results showed a statistically significant moderate correlation between the complexity index and the MUs for clinical as well as RapidPlan™ plans. In this work, it was revealed that optimizing VMAT H&N plans with RapidPlan™ does not increase the Mus nor the complexity of the plans, which is a positive outcome for plan deliverability.

It should be noted that this is the first study investigating the correlation between the MUs, MU factors, and 2D GPRs (%) with the complexity index in RapidPlan™ plans for H&N cases. This study confirms that plan complexity is affected by the MUs in both the RapidPlan™ and clinical plans for phases 54 Gy and 60 Gy. There was no correlation between the 2D GPRs (%) and the MUs, MU factor, or complexity for RapidPlan™ plans. On the contrary, a study focusing on prostate cancer cases, using an in-house KBP algorithm, observed that the 2D GPRs (%) were correlated weakly to moderately with complexity metrics [25]. Therefore, more research is needed to draw safe conclusions concerning the correlation of 2D GPRs (%)with plan complexity for KBP H&N treatment plans.

To our knowledge, this is the first study focusing on the evaluation of the plan deliverability of H&N RapidPlan™ models. Therefore, the current results may be comparable to studies referring to other treatment sites such as prostate cases. Tamura et al. found similar results for both PSQA outcomes (in terms of GPR) and MUs between clinical and RapidPlan™ plans for prostate cancer cases, an outcome which is in line with those of this study [26]. Hussein et al. did not find any statistically significant difference between RapidPlan™ and clinical plans in the MUs or the modulation complexity score [27] (similar to the complexity index used in this study) for prostate cancer cases, whereas RapidPlan™ plans had more MUs compared to the clinical ones for cervical cases [12]. Hundvin et al. showed that the MUs were similar between RapidPlan™ plans and clinical ones for prostate cancer cases [28]. On the other hand, Kubo et al. found increased MUs and more complicated MLC sequences compared to the clinical ones for prostate cancer patients [29]. Another study assessed the deliverability of VMAT plans for prostate cancer patients from three different institutions, and they concluded that, despite the statically significant differences in MUs or the modulation complexity score among the centers, the RapidPlan™ plans were deliverable [30].

### 4.3. Limitations and Future Directions

In terms of limitations, the training set size of the H&N 54 Gy, 60 Gy, and 70 Gy models (41, 43, and 40 plans, respectively) should be larger provided that these models are general scope and refer to larynx, nasopharynx, and oropharynx cases. The creation of a model which is specific requires a smaller training set compared to a general model, but specific models are susceptible to overfitting. Overfitting can lead to too-specific class solutions that exclude general cases. For instance, a specific larynx model could create optimal treatment plans for larynx cases, but it is likely to fail in an oropharynx case due to overfitting. The creation of a general model which could be implemented on a variety of treatment sites could be more practical for clinical routines. However, a general model requires a bigger training set using a suitable number of cases for each treatment site subgroup. There is one study which investigated the training set size requirements of the KBP models, and they concluded that 20 cases were enough to accurately predict the DVH for the rectum compared to the 75 cases required to predict the DVH for the bladder [31]. The training set size also depends on the number of available treatment plans for each treatment site in the department. Therefore, for either a general or specific-scope model, the results of the validation process will reveal if the model could be implemented in clinical settings.

In this study, the brain, eyes, optic chiasm, and optic nerves were OARs that remained untrained in all models, because the minimum of 20 treatment plans in which these OARs should belong in the in-field region was not reached. As a result, this issue may have an impact on nasopharynx cases, and the planner should pay attention and add manual optimization objectives for these structures. The training of the help structures was not considered, but might help the optimization process and the dose distribution. Moreover, we did not check the impact of multi-criteria optimization (MCO) in combination with RapidPlan™ because MCO was not available in our institution.

Concerning future directions, the training of the help structures, such as the ‘rings’ that aided the optimization process, could be performed. This may improve the quality of future RapidPlan™ models. The experience gained from this study will be useful for the creation of RapidPlan™ models for other treatment sites in our institution. Moreover, model creation which takes as an input plans generated from a RapidPlan™ model is a process that is described in the literature as ‘iterative learning’, and the results concerning plans’ quality are promising [28,32]. There are two studies available in the literature that combine the use of multi-criteria optimization (MCO) and RapidPlan™, and they are limited to one treatment site (H&N carcinoma) [9,33]. As a result, there is room to explore the combination of MCO with KBP for various treatment sites.

## 5. Conclusions

The training and validation process of H&N RapidPlan™ models is a time intensive process and needs attention to be safely introduced in a clinical setting. However, the results showed that there is a clinical benefit in terms of dosimetry, especially for the OARs, plan deliverability, and plan complexity with the use of RapidPlan™ for H&N cancer cases. In conclusion, knowledge-based treatment plans can be safely adapted into clinical routines, providing improved plan quality in a time-efficient way while minimizing user variability, creating a new standard for radiation oncology.

## Figures and Tables

**Table 1:** Success and failure rates for the open-loop and closed-loop validation sets.

Success and Failure Rates	Open-Loop	Closed-Loop
Success on 1st optimization	54.5%	80%
Success on 2nd optimization	90%	100%
Failure on 1st optimization	45.5%	20%
Failure on 2nd optimization	10%	0%

**Table 2:** Relative differences between clinical and RapidPlan™ plans of the low-risk PTV, intermediate-risk PTV, and high-risk PTV for the open-loop validation set.

PTV	Dosimetric Endpoint	Relative Difference $\left(\frac{clinical-RapidPlan}{clinical}\right)\times 100\%\;\mathrm{Mean}\;[\mathrm{Range}]\;(\%)$	*p*-Value
Low-risk PTV (54 Gy)	HI	−1.07 [−35, 29.9]	0.549
	CI	0.57 [−3.62, 5.76]	0.259
	$V_{107\%}$	2.34 [−4.9, 19.5]	0.082
Intermediate-risk PTV (60 Gy)	HI	−5.46 [−83.3, 57.1]	0.776
	CI	3.00 [−2.73, 11]	0.001 ^1^
	$V_{107\%}$	2.68 [−5.67, 12.9]	0.027 ^1^
High-risk PTV (70 Gy)	HI	−4.58 [100, 55.6]	0.358
	CI	−0.30 [−16.8, 4.86]	0.394
	$V_{107\%}$	−8.73 [−400, 100]	0.041 ^1^

^1^: statistically significant result (0.05 level of significance).

**Table 3:** Relative differences between clinical and RapidPlan™ plans of the 18 OARs for the open-loop validation set.

OAR	Dosimetric Endpoint	Relative Difference $\left(\frac{clinical-RapidPlan}{clinical}\right)\times 100\%\;\mathrm{Mean}\;[\mathrm{Range}]\;(\%)$	*p*-Value
Brainstem	Dmax ^1^	14.1 [−3.19, 40.6]	0.001 ^2^
PRV ^6^ Brainstem	Dmax ^1^	10.7 [−7.83, 38.6]	0.001 ^2^
Esophagus	$V_{45\mathrm{Gy}}$ ^4^	16.6 [−350, 78.8]	<0.001 ^2^
Lens L	Dmax ^1^	16.7 [−7.56, 66.7]	0.069
Lens R	Dmax ^1^	17.8 [−1.53, 65.5]	0.036 ^2^
Lips	Dmean ^3^	10.2 [−71.4, 38.7]	0.006 ^2^
Mandible	Dmax ^1^	4.58 [−6.14, 21]	0.003 ^2^
Optic Chiasm	Dmax ^1^	−6.5 [−20.4, 28.7]	0.063
Oral cavity	Dmean ^3^	5.32 [−22.7, 15.9]	0.064
Parotid L	Dmean ^3^	9.07 [−32.4, 37.3]	0.030 ^2^
Parotid R	Dmean ^3^	8.51 [−26.7, 33]	0.013 ^2^
Pharyngeal Constrictors	Dmean ^3^	4 [−10.2, 10.5]	0.007 ^2^
Spinal Cord	Dmax ^1^	5.9 [−21.8, 35.5]	0.063
PRV ^6^ Spinal Cord	Dmax ^1^	3.69 [−23.8, 26]	0.099
Spinal Canal	Dmax ^1^	2.57 [−20.5, 21.4]	0.279
Submandibular gland L	Dmean ^3^	−5.37 [−63.8, 10.3]	0.476
Submandibular gland R	Dmean ^3^	−6.94 [−55.7, 8.84]	0.904
Thyroid	$V_{26\mathrm{Gy}}$ ^5^	1.93 [0, 14.9]	0.018 ^2^

^1^: Dmax is the maximum dose received by the OAR. ^2^: Statistically significant result (0.05 level of significance). ^3^: Dmean is the mean dose received by the OAR. ^4^: $V_{45\mathrm{Gy}}$ is the volume of the esophagus receiving 45. ^5^: $V_{26\mathrm{Gy}}$ is the volume of the thyroid receiving 26 Gy. ^6^: PRV is the volume of the planning organ at risk.

**Table 4:** Mean 2D gamma passing rates ($\bar{\mathrm{GPR}\left(\%\right)}$) of the clinical and RapidPlan™ plans in the open-loop validation set.

Phase	Criterion	$\bar{GPR\left(\%\right)}\pm SD\;\mathrm{Clinical}$	$\bar{GPR\left(\%\right)}\pm SD\;\mathrm{RapidPlan}\text{™}$	$\bar{\Delta GPR\left(\%\right)}$	*p*-Value
54 Gy	3%/3 mm	99.4 ± 0.6	99.3 ± 0.8	0.07	0.962
	3%/2 mm	98.6 ± 1.4	98.9 ± 1.3	−0.29	0.314
	2%/3 mm	98.5 ± 1.6	98.2 ± 1.8	0.28	0.856
60 Gy	3%/3 mm	98.9 ± 1.1	99.3 ± 0.6	−0.31	0.231
	3%/2 mm	97.6 ± 1.9	98.6 ± 1.1	−0.95	0.027 ^1^
	2%/3 mm	97.3 ± 1.7	98.3 ± 1.1	−0.93	0.018 ^1^
70 Gy	3%/3 mm	98.0 ± 1.7	99.0 ± 1.0	−1.07	0.038 ^1^
	3%/2 mm	96.1 ± 3.1	98.2 ± 1.7	−2.12	0.019 ^1^
	2%/3 mm	96.3 ± 2.6	97.8 ± 1.7	−1.51	0.048 ^1^

^1^: statistically significant result (0.05 level of significance).

**Table 5:** Monitor units (MUs), modulation factor (MU factor), and the complexity index for clinical plans against RapidPlan™ plans in the open-loop validation set.

Parameter	Number of ARCs	Phase	Clinical	RapidPlan™	*p*-Value
MU	47	54 Gy	243 ± 112	240 ± 110	0.525
	34	60 Gy	321 ± 153	322 ± 146	0.784
	28	70 Gy	373 ± 165	360 ± 151	0.577
	109	total	300 ± 149	296 ± 141	0.469
MU factor	47	54 Gy	4.49 ± 2.07	4.44 ± 2.03	0.525
	34	60 Gy	53.5 ± 25.6	53.6 ± 24.3	0.778
	28	70 Gy	37.3 ± 16.5	36.0 ± 15.1	0.577
	109	total	28.2 ± 27.1	27.9 ± 26.5	0.394
complexity index [mm^−1^]	47	54 Gy	0.120 ± 0.028	0.120 ± 0.023	0.498
	34	60 Gy	0.132 ± 0.029	0.138 ± 0.032	0.135
	28	70 Gy	0.152 ± 0.029	0.153 ± 0.029	0.785
	109	total	0.132 ± 0.031	0.134 ± 0.031	0.185

## Data Availability

The data presented in this study can be found here: https://data.mendeley.com/v1/datasets/hdhrnsxxvh/draft?preview=1 (accessed on 4 February 2023).
